# Serum Parathyroid Hormone Is a New Potential Risk Factor in Multiple Myeloma

**DOI:** 10.1155/2014/804182

**Published:** 2014-05-19

**Authors:** Min-Gu Kang, Eun-Jeong Won, Hyun-Woo Choi, Hye-Ran Kim, Hyun-Jung Choi, Hye-Ri Park, Jong-Hee Shin, Soon-Pal Suh, Dong-Wook Ryang, Myung-Geun Shin

**Affiliations:** ^1^Department of Laboratory Medicine, Chonnam National University Medical School and Chonnam National University Hwasun Hospital, 160 Ilsim-ri, Hwasun-eup, Hwasun-gun, Jeollanam-do 519-809, Republic of Korea; ^2^Laboratory of Metabolism, National Cancer Institute, National Institutes of Health, Bethesda, MD, USA; ^3^Brain Korea 21 Plus Project, Chonnam National University Medical School, Gwangju, Republic of Korea

## Abstract

We hypothesized that serum PTH might be associated with various clinicopathological parameters in multiple myeloma (MM). So we investigated the implications of serum PTH in MM patients and the relationship with other risk factors of MM. A total of 115 patients who were newly diagnosed with MM were enrolled. Serum PTH level was 24.7 ± 34.9 (ranged 0.0–284.1) pg/mL. Serum levels of IgG, IgM, FLC-lambda, albumin, and LDH were in positive correlation with serum PTH. Compared to non-high PTH (<68.3 pg/mL) group, the hazard ratio (HR) for overall survival was higher for group with high PTH level (≥68.3 pg/mL) (HR, 1.710). Furthermore, the patient group with high PTH level showed inferior progression-free survival than non-high PTH group (*P* = 0.056). Interestingly, subgroup analysis showed that serum PTH level at diagnosis was associated with risk factors and clinical outcome in MM patients, especially in complete remission group, transplantation cases, ISS stage II cases, and cases without chromosome abnormality. In conclusion, this study showed that blood PTH level in MM at diagnosis was associated with risk factors and clinical outcome in MM patients.

## 1. Introduction

Parathyroid hormone (PTH) is synthesized and secreted by the chief cells of the parathyroid gland. PTH has a positive impact on hematopoietic stem cells by indirectly decreasing hematopoietic cell apoptosis and is currently being investigated as a potential therapeutic remedy to stimulate hematopoiesis and enhance bone marrow engraftment [1–4].

According to the previous study [5], it was hypothesized that elevated PTH may mediate the induction of multiple myeloma (MM) through the downstream biologic effects of interleukin 6 (IL-6). Meanwhile, PTH stimulates stromal-osteoblastic cells to secrete IL-6 [6]. Because this cytokine plays a key role in the development of plasma cell dyscrasias [7], high PTH levels may facilitate the emergence and growth of a plasma cell clone [8].

However, there were a limited number of studies regarding the relationship between serum PTH level and its clinicopathological implications in MM until now. Additionally, pathobiology of MM suggests that serum PTH level might be associated with clinical consequences of MM patients. So we addressed the possible relationship between serum PTH level and pathophysiology of MM patients with other various clinical parameters. This study presents data which showed the prognostic implications of serum PTH in MM patients and their relationship with other risk factors of MM.

## 2. Materials and Methods

A total of 115 patients who were newly diagnosed with MM were enrolled between 2006 and 2012. MM was diagnosed based on the clinical, laboratory, and radiologic findings. The details of diagnostic criteria were as follows: monoclonal protein in serum or urine, bone marrow clonal plasma cells or plasmacytoma, and the evidence of related organ or tissue impairment (hypercalcemia, renal insufficiency, anemia, and bone lesions).

Serum PTH level was measured at the time of the diagnosis by automated 2-site chemiluminescent microparticle immunoassay of AxSYM system (Abbott Diagnostics, IL, USA) according to the manufacturer's instructions.

Chromosome analysis was performed on G-banded preparations from 48 hour bone marrow cell cultures without adding mitogens. The chromosome aberrations were described according to the International System for Cytogenetic Nomenclature 2005 and 2009. In additional to conventional cytogenetic analysis, fluorescent in situ hybridization (FISH) was applied in appropriate bone marrow specimens using 13q14.3, 13q24, and 17p13.1 (p53) deletion probes and 14q32 (IGH) break apart probe (Vysis, Des Plaines, IL, USA) according to the manufacturer's instructions.

In addition to serum PTH, other clinical parameters were reviewed for age, sex, plasma cell percentage in bone marrow, serum monoclonal protein, immunoglobulin (Ig) level, free light chain (FLC)—kappa and lambda, FLC ratio, calcium, creatinine, hemoglobin (Hb), albumin, beta-2 microglobulin, lactate dehydrogenase (LDH), international staging system (ISS) stage, international myeloma working group (IMWG) response, chromosome abnormality, bone lesion, treatment outcome, and so forth. The diagnostic criteria of MM, ISS stage, and the international myeloma working group (IMWG) response were based on the precedent review [9].

According to the serum PTH level at the time of MM diagnosis (cut-off; 68.3 pg/mL, reference range; 15–68.3 pg/mL), the study population was divided into non-high PTH group and high PTH group. Then, previously reviewed parameters of MM patients were compared between the two groups. In addition, the correlations of serum PTH level with other laboratory parameters of MM patients were examined.

The collected data were analyzed by PASW version 18.0 (SPSS Inc., Chicago, IL, USA). In detail, Spearman's correlation analysis was performed to evaluate the association between various laboratory parameters with serum PTH level. Pearson's chi-square test or Fisher's exact test was performed to calculate the significance of association between PTH group and other parameters of discrete categorical variables including gender and IMWG response. Kruskall Wallis test or Mann Whitney *U* test was used to compare serum PTH level according to categorical classification such as IMWG response or monoclonal protein subtype. Additionally, Mann Whitney *U* test was performed to compare continuous variables such as age and laboratory data between non-high PTH and high PTH group.

Time dependent Cox regression analysis was used to dissect the individual impacts of prognostic factors of overall survival (OS) of MM patients. Kaplan-Meier estimation was used to plot survival curves, and log-rank tests were used to calculate the difference of OS and progression-free survival (PFS) between high PTH group and non-high PTH group. All tests were two-tailed and a *P* value of less than 0.05 was considered statistically significant.

## 3. Results and Discussion

Serum PTH level of 115 myeloma patients was 24.7 ± 34.9 (ranged 0.0–284.1) pg/mL. The reference range of serum PTH was 15–68.3 pg/mL in our laboratory. Of the various laboratory data of MM patients, higher levels of bone marrow plasma cell percentage, monoclonal protein concentration, creatinine, beta-2 microglobulin, and lactate dehydrogenase were seen in high PTH group rather than non-high PTH group (Table 1). And calcium level was significantly different (*P* = 0.016, Figure 2(a)) by the comparison of laboratory data (continuous variables) between non-high PTH group and high PTH group (cut-off PTH level = 68.3 pg/mL, Mann Whitney *U* test).

Previously, Arnulf et al. [8] reported that the prevalence of monoclonal gammopathy was high in patients with primary hyperparathyroidism compared to general population and that high PTH levels might facilitate the emergence of a plasma cell clone. This is consistent with above findings of higher levels of plasma cell percentage and monoclonal protein concentration in high PTH group.

On the other hand, male patient case, case with age of above 50 years, IgG kappa monoclonal protein type, bone lesion case, ISS stage III, and progressive disease case (by IMWG response criteria) occupied the main portion in both non-high PTH group and high PTH group (Table 1). Of the various clinical parameters, gender factor (male or female) revealed significant difference between non-high PTH group and high PTH group (*P* = 0.017) (Table 1).

**Figure 1:**
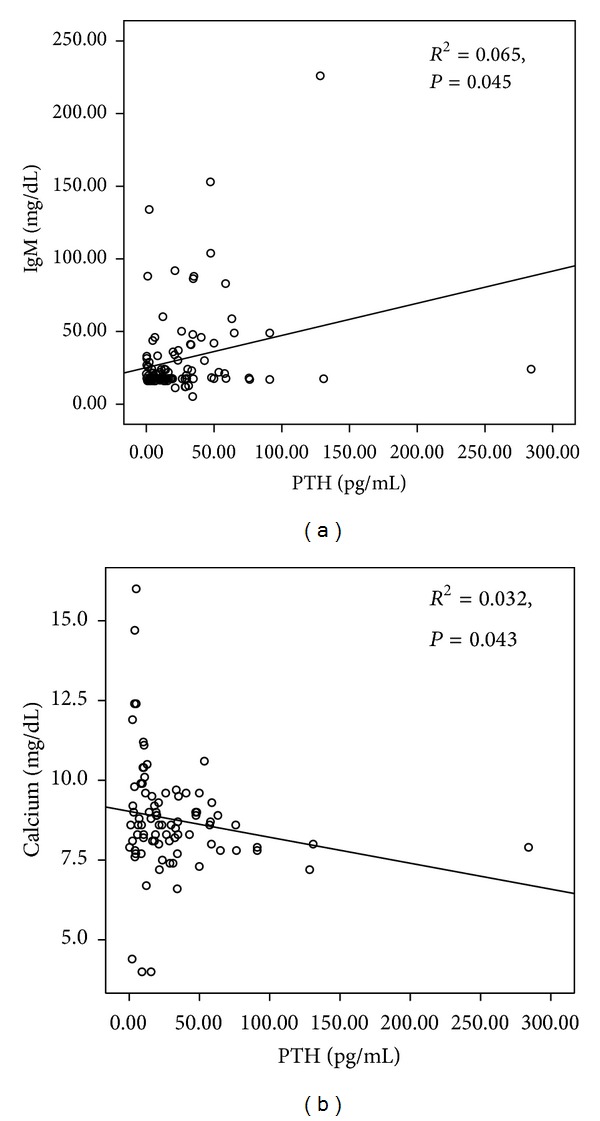
The correlations of PTH values with IgM (a) and calcium (b). Out of many other results of laboratory test performed for multiple myeloma patients, IgM (rho = 0.190, *P* = 0.045) and calcium (rho = −0.220, *P* = 0.043) showed meaningful correlation with serum PTH.

**Figure 2:**
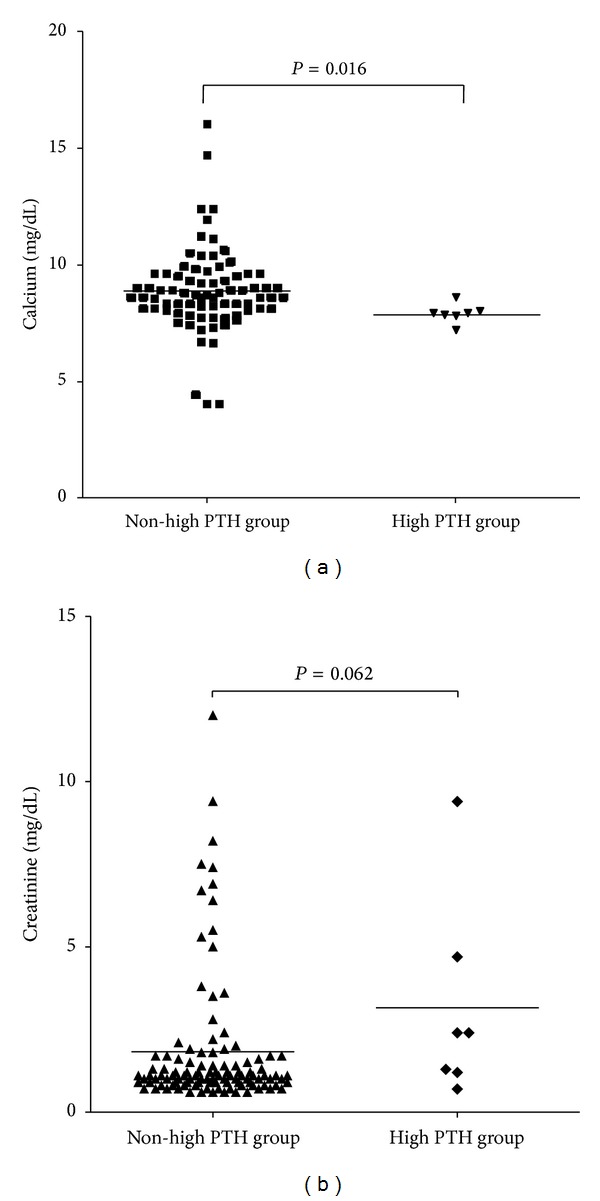
The comparison of calcium (a) and creatinine (b) level between non-high PTH group and high PTH group. Among the various clinical parameters of multiple myeloma patients, calcium (*P* = 0.016) and creatinine (*P* = 0.062) revealed moderate difference depending on the PTH level.

The serum levels of IgG, IgM, FLC-lambda, albumin, and LDH were in positive correlation with serum PTH. However, age, plasma cell percentage, monoclonal protein, IgA, FLC-kappa, FLC ratio, calcium, creatinine, Hb, and beta-2 microglobulin showed negative correlation with PTH (detailed data not shown). Among those above, IgM (rho = 0.190, *P* = 0.045) and calcium (rho = −0.220, *P* = 0.043) revealed statistically significant correlation with serum PTH (Figure 1). PTH may mediate the induction of multiple myeloma. Also, PTH can interact with other various chemokines, ligands, or hematopoietic niche. This process might influence the correlation. However, in terms of pathogenesis, the direct effect or meaning of correlation of PTH with IgM or calcium needs to be determined through further study.

In addition, serum PTH level in MM patients was not significantly different according to IMWG response (*P* = 0.450), ISS stage (*P* = 0.414), monoclonal protein subtype (*P* = 0.572), FISH result (*P* = 0.105), and chromosome analysis result (*P* = 0.353). Meanwhile, there was no significant difference of PTH according to gender (*P* = 0.250), age (above 50 years or below 50 years, *P* = 0.423), existence or nonexistence of clinical events including death (*P* = 0.571), disease progression (*P* = 0.322), bone lesion (*P* = 0.207), transplantation (*P* = 0.233), and chromosome abnormality (*P* = 0.124).

Then again, seven patients out of total 115 multiple myeloma patients did not receive treatment. Among 108 patients who were treated, 76 patients (70.4%) underwent a single set of treatment, while 32 patients (29.6%) were treated by multiple sets serially. In detail, CTD (cyclophosphamide, thalidomide, and dexamethasone) was the basic treatment modality in 48 patients, VCD (vincristine, cyclophosphamide, and dexamethasone) in 22 patients, MP (melphalan and prednisone) in 14 patients, and dexamethasone in 13 patients. And the therapeutic modalities were not significantly different between non-high PTH group and high PTH group.

According to Pirih et al. [1], PTH acts on bone marrow stromal cells to stimulate IL-6 production. Then, IL-6 synergizes with fms-like tyrosine kinase 3 ligand (Flt-3L), and it increases hematopoietic cell numbers. Namely, PTH acts with Flt-3L to maintain hematopoietic cells by limiting apoptosis. Also, Shiozawa et al. [10] showed that increasing the number of hematopoietic stem cell niche with PTH promoted metastasis, which means that hematopoietic stem cell niche served as a specific site where disseminated cells from original cancer gain footholds in the bone marrow.

Furthermore, the role of the bone marrow microenvironment in MM has been extensively studied in many models. The bone marrow provides signals that influence the behavior of MM cells (e.g., tumor cell growth, survival, and migration). And the cellular elements of the bone marrow (e.g., mesenchymal stem cells, osteoclasts, osteoblasts, and vascular endothelial cells) interact with MM cells directly or indirectly through secretion of stimulatory cytokines and chemokines such as IL-6 that induces survival, growth advantage, and drug resistance of MM [11, 12].

**Figure 3:**
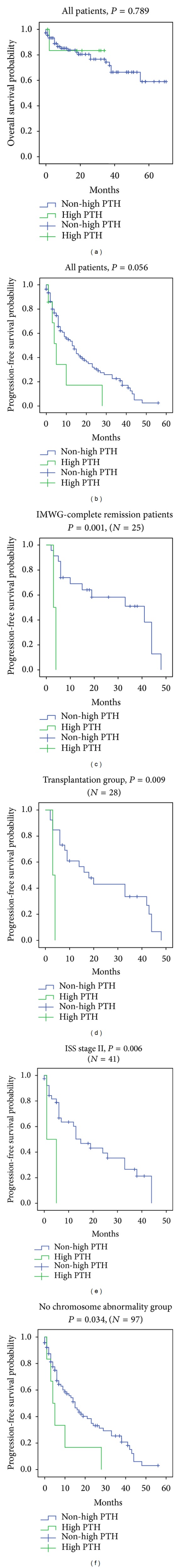
Impact of PTH on clinical outcome. Kaplan-Meier curves of overall survival (OS) and progression-free survival (PFS) stratified by PTH level for all patients (a-b), PFS of patients on complete remission state at the end-point of present study (c), PFS of patients with ISS stage II (d), PFS of patients with no chromosome abnormality (e), and PFS of patients who have undergone stem cell transplantation (f).

As the precedent study and reports mentioned above, the present study shows interesting results as well. Compared to non-high PTH (<68.3 pg/mL) group, the hazard ratio (HR) for overall survival was higher for the group with high PTH level (≥68.3 pg/mL) (HR, 1.710; *P* = 0.766; 95% confidence interval, 0.050~58.212) (Table 2). With regard to the prognostic implication of serum PTH value, the high PTH group (≥68.3 pg/mL) showed moderate, more, inferior PFS than non-high PTH group (median, 5 months versus 13 months, *P* = 0.056; Figure 3(b)).

By contrast, Pennisi et al. [13] reported that* in vivo* PTH treatment indirectly attenuated MM progression by stimulating osteoblastogenesis and increasing osteoblast production of antimyeloma factors and by minimizing oxidative stress and inflammatory conditions in myelomatous bone. On the other hand, PTH receptors were not expressed by myeloma cells and PTH had no effect on myeloma cell growth* in vitro*. Therefore, it seems that the clinical and prognostic implication of serum PTH level in MM patients still remains a controversial topic.

Nevertheless, present study was not based on experimental findings but on the clinical and practical data of MM patients. In addition, following results of the present study are consistent with the report of Pirih et al. [1].

Although no OS differences were found between high PTH group and non-high PTH group (Figure 3(a)), subgroup analysis revealed that the patients with high serum PTH level significantly had an inferior PFS than those with non-high serum PTH level. In detail, the subgroup analysis of PFS (high PTH group versus non-high PTH group) were as follows: patients (*N* = 25) who reached the complete remission (CR) state defined by IMWG response criteria at the end of the follow-up period (median, 3 months versus 41 months, *P* = 0.001; Figure 3(c)), patients (*N* = 28) who have undergone transplantation (median, 3 months versus 18 months, *P* = 0.009; Figure 3(d)), patients (*N* = 41) who belonged to ISS stage II (median, 1 month versus 15 months, *P* = 0.006; Figure 3(e)), and patients (*N* = 97) with no chromosome abnormalities (median, 4 months versus 15 months, *P* = 0.034, Figure 3(f)).

## 4. Conclusion

Serum PTH level at diagnosis was associated with risk factors and clinical outcome in MM patients, especially in CR group (IMWG response), transplantation case, ISS stage II, and case without having chromosome abnormality.

## Figures and Tables

**Table 1:** Baseline characteristics in MM patients with non-high and high PTH level.

	Non-high PTH group (*N* = 108, 93.9%)	High PTH group (*N* = 7, 6.1%)	*P*
Gender*			0.017
Male	57 (52.8)	7 (100.0)	
Female	51 (47.2)	0 (0.0)	
Age*			0.351
>50 years	96 (88.9)	7 (100.0)	
≤50 years	12 (11.1)	0 (0.0)	
Mean survival (months)	51	29	0.789
Laboratory data^†^			
PTH (pg/mL)^‡^	12.5 (0.0–64.9)	91.1 (75.8–284.1)	0.000
Bone marrow-plasma cell (%)	32 (5–90)	44 (24–97)	0.184
Monoclonal protein (g/dL)	4.1 (0.0–8.7)	5.1 (1.9–6.2)	0.539
IgG (mg/dL)	2400 (151–80000)	772 (147–8430)	0.819
IgA (mg/dL)	48.0 (7.0–8630.0)	48.0 (10.0–8750.0)	0.593
IgM (mg/dL)	17.9 (5.3–153.0)	18.0 (17.0–226.0)	0.761
FLC-kappa (mg/L)	32.9 (0.3–62500.0)	27.0 (1.0–67.0)	0.298
FLC-lambda (mg/L)	20.0 (1.0–33900.0)	29.2 (2.8–867.0)	0.734
FLC ratio	2.2 (0.0–13242.4)	0.6 (0.0–16.3)	0.555
Ca (mg/dL)	8.7 (4.0–16.0)	7.9 (7.2–8.6)	0.016
Cr (mg/dL)	1.1 (0.6–12.0)	2.4 (0.7–9.4)	0.062
Hb (g/dL)	9.4 (5.0–15.0)	10.1 (5.7–13.2)	0.734
Albumin (g/dL)	3.1 (1.1–5.1)	3.5 (2.4–4.0)	0.721
*β*2-MG (*μ*g/L)	3981.5 (620.4–80000)	7522.0 (2078.0–33035.5)	0.342
LD (IU/L)	356.0 (85.0–1113.0)	364.0 (239.0–714.0)	0.595
Monoclonal protein type*			0.932
IgG*κ*	36 (33.3)	2 (28.6)	
IgG*λ*	27 (25.0)	1 (14.3)	
IgA*κ*	20 (18.5)	2 (28.6)	
IgA*λ*	11 (10.2)	1 (14.3)	
Free *κ*	4 (3.7)	0 (0.0)	
Free *λ*	9 (8.3)	1 (14.3)	
Not available	1 (0.9)	0 (0.0)	
Bone lesion case*	76 (70.4)	5 (71.4)	0.953
Transplantation case*	26 (24.1)	2 (28.6)	0.788
Chromosome abnormality case*	17 (15.7)	1 (14.3)	0.918
ISS^∗§^			0.685
Stage I	25 (23.1)	1 (14.3)	
Stage II	39 (36.1)	2 (28.6)	
Stage III	44 (40.7)	4 (57.1)	
IMWG response*			0.710
CR	23 (21.3)	2 (28.6)	
VGPR	8 (7.4)	1 (14.3)	
PR	17 (15.7)	0 (0.0)	
Progressive disease	47 (43.5)	3 (42.9)	
Stable disease	6 (5.6)	1 (14.3)	
Not available	7 (6.5)	0 (0.0)	

*Number of patients (%). ^†^Median (range).

^‡^The reference range of PTH was 15–68.3 pg/mL. We have classified patient cohorts into non-high PTH group and high PTH group based on this criteria.

^§^International staging system: I, *β*2-MG <3500 *μ*g/L and albumin ≥3.5 g/dL; II, not fitting stage I or II; stage III, *β*2-MG ≥5500 *μ*g/L.

PTH: parathyroid hormone; FLC: serum free light chain; Ca: calcium; Cr: creatinine; Hb: hemoglobin; *β*2-MG: beta-2 microglobulin; LD: lactate dehydrogenase; *κ*: kappa; *λ*: lambda; ISS: international staging system; IMWG: international myeloma working group; CR: complete response; VGPR: very good partial response; PR: partial response.

**Table 2:** Time-dependent Cox regression analysis for the overall survival in MM patients.

Factors	Hazard ratio	95% CI	*P*
PTH			
≥68.3	1.710	0.050~58.212	0.766*
<68.3	1.0 (reference)		
Age	1.004	0.952~1.058	0.894
Sex			
Male versus Female	1.294	0.422~3.968	0.652
ISS stage			
I versus II	0.138	0.015~1.250	0.078
I versus III	0.171	0.017~1.683	0.130
Albumin	0.136	0.038~0.491	0.002^†^
*β*2-MG	1.0	1.000~1.000	0.963
Calcium	1.271	0.965~1.675	0.088
Creatinine	1.126	0.735~1.725	0.586
Hemoglobin	1.097	0.824~1.462	0.526
FLC ratio	1.0	1.000~1.000	0.030^†^

*All parameters considered; the overall *P* value of PTH was 0.557.

^†^Statistical significance.

PTH: parathyroid hormone; FLC: serum free light chain.
